# A Qualitative Study of the Mistreatment of Medical Students by Their Lecturers in Polish Medical Schools

**DOI:** 10.3390/ijerph182312271

**Published:** 2021-11-23

**Authors:** Marta Makowska, Joanna Wyleżałek

**Affiliations:** Department of Sociology, Institute of Sociological Sciences and Pedagogy, Warsaw University of Life Sciences, 02-787 Warsaw, Poland; joanna_wylezalek@sggw.edu.pl

**Keywords:** medical students, abuse, mistreatment, Poland

## Abstract

Objective: To describe experiences of mistreatment among Polish medical students. Methods: Nine focus groups were carried out with 92 students from three medical universities in Poland (in Gdansk, Krakow, and Warsaw). Results: The mistreatment of medical students included verbal abuse, disregard, and obstacles to pass exams. Students experienced humiliation, belittlement, insults, criticism, shouting, and indecent comments. The lecturers did not respect the students’ time; they did not show understanding for their absences; sometimes, they came to class unprepared while other times, they showed indifference regarding the well-being of students. Respondents stated that they were given enormous amounts material such that they found it far beyond their ability to learn; they were not given information about which textbooks were to be used; exams were incredibly detailed and difficult; and the grading system was unfair. In general, most students did not report the mistreatment. The respondents noticed the negative consequences of their mistreatment, which included a decrease in self-esteem and increased levels of anxiety and stress. This may translate into a lack of empathetic approach to patients. Conclusions: The phenomenon of the mistreatment of medical students requires more attention in Poland. It is important to raise awareness of the significant consequences of this.

## 1. Introduction

Medical education is designed to shape students into doctors who care about the well-being of their patients and who will be reliable and trustworthy. The medical profession, like many other service-related occupations, is a profession with a social mission and is associated with social trust [1]. Accordingly, doctors are responsible not only for the health and life of patients but also for maintaining the culture of trust in social relations. The physician must act in accordance with the values and norms instilled during professional training, they must use their medical knowledge in a manner that considers their patients’ best interests, and they should promote good public health practices. However, there are many obstacles on the path to becoming a physician.

For many students, beginning medical studies is a dream realised and many choices over the course of their lives have been in preparation for this [2,3,4]. The majority of students are motivated to go to medical school due to their inclination to help others [2,4,5,6,7]. Other reasons for choosing a medical education include, the respect/prestige that comes with being a doctor [4,5,7], an interest in science/field [3,4,5], the desire for intellectual stimulation [6], the influence of family and friends or a family tradition [3,7,8], wanting a decent and interesting career [3], and the potential for a sizeable income [4,7]. “Most students embark on their professional education with excitement, idealism, and commitment but emerge disappointed with the process of becoming a physician” [9] (p. 572). The transformation of an idealistic scholar into a cynical student and then into an uncaring physician has been well described in the sociological literature [10,11,12]. This happens, inter alia, because students are overwhelmed by the large quantities of information they have to acquire and deal with medical uncertainty [12,13,14]. Students also become disheartened because medical school is focused more on scientific facts and theory and less on practical knowledge [14]. Additionally, students sometimes have to deal with misfortune and death [12,14] and they are also mistreated [12,15,16,17,18]. By definition, mistreatment: “arises when behaviour shows disrespect for the dignity of others and unreasonably interferes with the learning process” [18] (p. 706).

In the 1960s, Becker et al. noticed the problem of the mistreatment of medical students [12]. However, the current and ongoing academic discussion regarding mistreatment was brought to the fore by Silver in his 1982 article whereby he compared medical students to abused children. He noticed that shortly after starting their studies, students “become cynical, some dejected, others frightened or depressed, and a few filled with frustration” [15] (p. 309), which was the result of the abuse they experienced that largely went ignored. His observations were subsequently confirmed by numerous studies [16,17,18,19,20,21,22,23,24,25,26].

Mistreatment has serious negative consequences that can continue long after the student leaves university as it can affect their attitude towards both patients and colleagues. According to the literature, there are several consequences of mistreatment such as depression [21,23,24,25], anxiety [21,25], stress [16], anger [23,24], escapist and problem-related drinking [25], burnout [26], and lack of confidence [24,27]. Given these consequences, it is important to raise awareness of the enormous harm that can arise due to mistreatment.

Mistreatment, like many other institutional problems, is not a problem only at the individual level; it is often systemic and widespread. In some professions, severe initiation practices are the norm and there is often silent consent or no reaction to the abuse [28]. This, therefore, indicates that there is a problem of social tolerance of this kind of mistreatment. Social tolerance can differ according to historical and geopolitical conditions and according to the organizational management model. It is worth mentioning that organizational pathologies occur in every social environment, but the dynamics of introducing formalised methods of preventing and eliminating problems in organizations depends on the level of involvement of democratic institutions. Therefore, recognising the existence of medical student abuse in different social contexts is extremely important. When this occurs, the appropriate preventive steps can be taken at the institutional level (e.g., training staff to avoid mistreatment or creating appropriate guidelines and methods of reporting abuse).

The problem of the mistreatment of medical students is an international problem and has been reported in many different countries, including the United States of America, Canada, Japan, Finland, Mexico, New Zealand, Chile, and Argentina [29]. However, no in-depth research from Poland has been published so far on this subject, and what exists only briefly touches upon the problem [30]. However, Polish research examined medical student burnout [31] and also looked into the stress that medical students encounter and their use of psychologists to help deal with these issues. [32]. In Poland, a medical education lasts six years (12 semesters) and the number of class hours, including apprenticeships, cannot be less than 5700 h [33]. Medical education begins right after high school. The state sets admission limits for this field of study every year and only the best students (with higher grades) are admitted. Medical studies are usually full-time and free, although medicine can also be studied in Poland part-time-paid [34].

The following article will describe incidents of mistreatment experienced by Polish medical students. It aims to answer three research questions: (1) What kinds of mistreatment do Polish medical students experience? (2) How do Polish medical students react to this mistreatment? (3) What are the consequences of mistreatment that can be identified from discussions held with medical students?

## 2. Materials and Methods

Nine focus groups were carried out in three different cities in Poland—Gdansk, Warsaw, and Krakow. Ninety-two medical students (40 men and 52 women) took part. Seven were in second year, 41 in third year, 20 in fourth year, 11 in fifth year, and 13 in sixth year. Thirty-nine students were from Gdańsk, 27 from Krakow, and 26 from Warsaw. A few respondents were not native Polish; however, they spoke the Polish language very well. Information on whether or not the students paid for their studies was not collected. Each focus group had between 7 and 13 participants and included students from different years of medical school. Sample selection and focus group composition are described in more detail in an article previously published from this research [35].

The study did not obtain ethical consent because, at the time of the study, the university where the researchers worked did not have an ethics committee for social sciences. It should be noted that although the university did not have any ethics review procedures in place, the recruiting company ensured that the participants’ data were kept confidential and anonymous from the principal investigator (MM). Through the recruiting company, all respondents gave their informed consent to participate in the focus group interviews and were informed about their option to withdraw from the study at any time. The participants received a small remuneration from the recruiting company (PLN 150/Euro 35). The interviews transcripts from the focus groups and the script in Polish are publicly available in the Repozytorium Danych Społecznych on the website: https://rds.icm.edu.pl/dataset.xhtml?persistentId=doi:10.18150/ UFEA7T (accessed on 20 November 2021).

All focus groups lasted approximately two hours and were conducted in a professional focus group interview studio. The same interview script was used for all groups. The principal investigator was also the focus group facilitator. The primary purpose of the study was to identify the presence of pharmaceutical companies in the socialisation processes associated with the medical profession [35]. The issue presented in this article, the mistreatment of medical students, is a secondary thread that appeared during the discussion regarding the advantages and disadvantages of studying medicine. The problem of the mistreatment of students was recognised retrospectively and the thematic codes were generated inductively. The study was conducted within the theoretical framework of social constructivism. A thematic analysis was performed in line with the steps proposed by Braun and Clarke [36].

As a result, three main themes were generated: (1) mistreatment, (2) reaction, and (3) consequences. The mistreatment theme has three subthemes: (a) verbal abuse, (b) disregard, and (c) making it difficult to pass.

## 3. Results

### 3.1. Mistreatment

#### 3.1.1. Verbal Abuse

In all of the focus groups, in each city, there were some utterances that were classified under the theme of verbal abuse. Students said that they felt belittled and humiliated and that some of the faculty did not treat them with respect.

For the quotes that follow, please see the following explanations. Male—M; female—F. The student’s year of study is indicated in parentheses. Group—GP, followed by its number. The text in square parentheses was added by the authors for a better understanding of the respondent’s statements.

F (IV) *( … ) it’s still ok when they say to all of us that we are hopeless ( … ). But for example, I also had a situation in the group that ( … ) a friend ( … ) failed ( … ) practical [exam] ( … ) the lecturer after a week ( … ) told him that he should quit studies because she sees that he is not going to be a good doctor, that he is hopeless, he doesn’t know ( … ) and he should quit studies as soon as possible ( … ) It was terrible, because not only did she say it in front of our entire group, but it was a year ago, and this friend of mine is still brooding about it, that she told him this and [he] all the time thinks about it.*

(Warsaw, GP 1).

Students also said that they were sometimes insulted by lecturers. Here is an example of such an incident:

F (III) *( … ) a friend was called a moron, although he was not prepared but it still does not give an excuse, it does not justify.*

(Krakow, GP 3).

Students spoke about being shouted at and blamed for minor offences. Some of them said that they have gotten used to it and that they no longer care when these incidents happen.

F (VI) *Sometimes in a ward, we just wait for someone to fuck us thoroughly. Because we’re in the wrong place, we’re standing, we went wrong, nobody told us and ...*

(Warsaw, GP 3).

The ability to withstand criticism is extremely valuable in medical studies. A common thread among some of groups indicated that it was advantageous to make yourself resilient and to stop worrying. But many indicated that this was difficult to do. Some students said that they were often afraid to speak up for fear of being criticised.

F (V) *Well, I was very often meet with aggression from the lecturers and with very strong criticism that we do not know something, that we are not prepared, that we did something wrong ( … ) We are being criticised for making mistakes, and then we go to the second class with the second assistant and we are freaked out that we are not attempting to express our opinion, but we are just traumatised after the previous class when we tried to speak up and learn something, and we got for it ( … ).*

(Gdansk, GP 1).

It was noted that sometimes lecturers behave in a way completely inappropriate for their position and that they make inappropriate comments.

M (III) *( … ) the lecturer gave such a distasteful comment [after the student’s presentation]. I quote that: “If physiology is a beautiful woman, it was you who raped her from two sides.” Literally. These types of statements.*

(Krakow, GP 3).

The disclosure of these occurrences was also accompanied by the feedback of some students who stated that such situations never happened to them, or that the students who explained these incidents may have been overreacting because, as they stated, verbal abuse happens everywhere, at every university.

M (III) *I’m still in my third year, that’s half of my studies ahead of me. But somehow, so far, I have not come across any such, let us say, a very severe form of criticism ( … ). It happened to me once that a lecturer ( … ) made an exceptional attempt to show me that it was very wrong, that I forgot ( … ) my apron. ( … ) She stopped after a while when I told her it was not pleasant, and I already know that I didn’t take that apron.*

(Gdansk, GP 1).

#### 3.1.2. Disregard

Disregard is a manifestation of the low position of the student in the medical hierarchy. Firstly, the students complained that the lecturers did not respect their time. Across all the cities, they spoke about waiting for classes to begin. However, despite the outrage of some of these students about this, there were also those who defended such behaviour—explaining that the lecturers (who are also often physicians) are very busy, and that the students are not the most important.

M (III) *When it comes to respecting time, for example, sometimes I would like too, if the lecturer knows that he will be late, because he cannot [come] earlier, I understand it, but I would want him ( … ) to let us know, so we do not sit for half an hour in the classroom and do not wait without any information.*

F (III) *Well, this is a question of whether it is a result of ill will, because, because those are students, or whether a person does not have time and is overworked. And he has more important things on his mind.*

(Krakow, GR 2).

Students also pointed out that lecturers are allowed to be late, but if a student is late, he or she will face negative consequences for it. Failure to attend a test, even with a proper excuse, may cause the student to be reprimanded by their teacher.

M (III) *For example, I remember the case of a colleague who was in a hurry to attend a test, but just somehow ran into the zebra crossing in such an unfortunate way that he caused an accident. ( … ) He didn’t do it on purpose because he did not want to come to the test, right? Anyway, a random event happens and there was a problem with it.*

F (III) *It often happens that lecturers are late for tests, for example, half an hour, they calmly climb stairs and no problem ( … ). But I remember ( … ) we entered the classes and were criticised for 15 min, and I remember the assistant ( … ) shouting at us that we were late for her classes, and we tried to explain to her that we just passed the test ( … ), and that we were not able to get out of it sooner. And I remember that the assistant said that she was not interested in it at all and then she decided to mark us as present on this class because at the beginning she put us absences, so it was terrible.*

(Krakow, GP 1).

Another example of the disregard for students occurs when some of the lecturers come to class but are not prepared. All they do is read the presentations aloud and, because of this, the students feel that they are wasting their time.

M1 (IV) *I don’t know if you have this feeling sometimes that, mmm, the classes are over, that I’m going home, finally I will be able to learn something (group laughter, many people nod).*

M2 (III) *Because lecturers don’t do anything to make it easier for us, right? They read presentations that are available on the faculty website, i.e., we can come and see someone reading from the monitor for 2 h.*

(Warsaw, GP 3).

Students in all three cities also said that they sometimes have to look for their clinical teachers because they intentionally do not show up for class. One of the students called it “a game of looking for an assistant” (Warsaw, GP 3).

M (III) *Also, [clinical] teachers, for example, can run away from students, hide in the hospital (group laughter). When students ask if there is such and such a doctor, this doctor replies—no.*

(Krakow, GP 3).

#### 3.1.3. Making It Difficult to Pass

The focus group participants often reported that there are too many students enrolled in medical studies; that there is not enough capacity. Therefore, there is enormous psychological pressure on the students because, eventually, some will have to drop out because of this lack of capacity. Usually, students drop out during the first year, although there were also third-year students who said that they were still afraid of whether they could hold on. The students also pointed out that it is not possible to repeat some of the subjects and even if they could, it would be very expensive to do so as the cost could range from several hundred to several thousand złotys while the gross average monthly salary in Poland in 2019 was PLN 4918. In many of the focus groups, students repeated the saying, “If you pass anatomy in the first year, you know that you will be a doctor, and if you pass pharmacology in the third year, you even know when”.

M1 (IV) *( … ) First year is to sow, because of course if you don’t know what’s going on, it’s about money. The student is expensive to the university, [they] have to kick them out, otherwise the university will get poorer ( … ).*

M2 (III) *( … ) First year is such a terrible, terrible psychological pressure on students, you can feel the lecturers’ breath on the back of your neck all the time, that the only thing they want is to throw you out of your studies. And no matter what you do, they won’t appreciate you or do anything for you.*

(Krakow, GP 3).

According to the vast majority of the students, tests are intentionally set with too many details and that this is done to rid the university of some of the students. These tests do not gauge relevant knowledge, they only test the ability to memorise.

M (III) *Especially there is such a phenomenon of artificial obstruction of passing subjects, i.e., questions are specifically asked in such a way to confuse the student a bit ( … )*

K (V) *Such as double denials ( … )*

Focus group facilitator: *Do you think they want you to fail?*

Group: *Yes, yes, yes (….).*

(Gdansk, GP 1).

Grading is done in such a way that the distribution of grades complies with the normal distribution, and not the actual knowledge of the students. This causes unhealthy competition and, as students say, is unfair.

F (III) *For example, many grading systems are based on the Gaussian curve, which would not be so bad if some students were not kind of stuck, that this ... is not ...*

M1 (IV) *We don’t want to, the departments don’t want to cut us anymore, so cut yourselves.*

M2 (III) *That, for example, the threshold for the grade, 4 in biochemistry was for 77 [and max was] 80 points. [The grade system in Poland is from 2 (fail) to 5 (very good)].*

(Krakow, GP 3).

Many students pointed out that due to unfairness, the amount of time they spend on learning does not directly translate into their grades. The quote below demonstrates this.

F (III) *So, in our year the anatomy was quite specific ( … ) over 100 people failed, and random people pass, depending on which turn exam was taken (….) a person who, for example, who was fourth in the Anatomical Olympics, did not pass ( … ). I felt such a great injustice, the department wanted to show that it was not entirely up to us, that they can do whatever they want…*

(Gdansk, GP 3).

Sometimes the desire to get good grades results in unusual situations that would not ordinarily be necessary, such as learning out-of-date information (because students know that they will be asked about it) or learning material with mistakes and even writing answers on exams that are not in line with their beliefs.

M1 (III) *Such an absurd situation, e.g., when someone gives us some knowledge and let’s assume that I do not agree with it, it’s like I have to write this on the exam, as there is no other option. I can’t say no, because then I won’t pass. As if there is no such chance of negotiating ( … ).*

M2 (VI) *Or now, now, for example, in the sixth year, we have e-learning exams ( … ) and there are errors in these tests ( … ). And you have to mark the wrong [answer], because if you mark the correct [answer] you have a lower grade.*

(Gdansk, Gr 2).

A very common problem was also that lecturers do not specify what information should be paid attention to nor which textbooks should be read in preparation for an exam, and the magnitude of the material often exceeds the ability to learn it all. The quote below demonstrates this.

M (III) *This is probably another problem, we do not have exactly what we are supposed to learn from, really, we have some different textbooks, but if we wanted to rewrite each of these books from cover to cover, one year will be not enough for one subject.*

(Krakow, GP 1).

### 3.2. Reaction

All the above-mentioned wrongdoings ought to be reported to the administrators of the universities. However, many students expressed their belief that reporting was not the solution, because, from their experience, it would not result in any changes. However, some respondents shared stories when the situation improved because of reporting.

F (III) *But with accusations and the effect of accusations, these are also different, different cases. Because we had, for example, a case in surgery clinics that the clinical teacher was not interested in us, someone told the professor that the clinical teacher was not interested in us and the professor said: ‘You have to take care of them, show them something. I know you don’t have time, but you have to find it for them.’ And he actually showed us something, so...*

M (VI) *Worse if the professor is ignoring us, and there is nobody over him.*

(Krakow, GP 2).

One of the students told a story about a lecturer intimidating students and that she would take revenge if anyone gave her negative feedback during the annual evaluations (students are given a survey about their lecturers at the end of the academic year).

F (IV) *And it is also necessary to take into account the fact that these surveys are supposedly anonymous, but for example we had a lecturer who, because they [lecturers] also read it, they also had access to these opinions about them, and she mentioned that ( … ) someone wrote this and this about her, and she ( … ) said that she knew exactly which student wrote it and she had to get her.*

(Warsaw, GP 1).

### 3.3. Consequences

Mistreatment has psychological consequences for students. Students pointed out that it is difficult to maintain a high self-esteem if you are constantly humiliated. They also experienced anxiety, such as the fear of being thrown out of their studies, as described earlier.

F (V) *Even the nurses treat us as like an unwanted mongrel. Really, it hits your self-esteem a lot. Such criticism is terribly discouraging.*

(Gdansk, GP 1).

Students often said that medical studies are very stressful and that it can be difficult to deal with this. Across all universities in this study, the most feared exam reported was the anatomy exam taken during the first year. Many report that it leaves a mark on the psyche for a very long time. Practical exams on this subject are designed in such a way that students enter the room where the cadaver is located and there are several anatomical structures marked. The students have a very small amount of time to write the name of the structure in Polish, Latin or English. When the time has finished, a bell or buzzer alarms and this means that you must move on to the next room. This sound paralyses many students.

K1 (III) *( … ) In the Lajkonik [restaurant], when [sandwich] heats up (….) there is an identical sound (….) it actually works for everyone, everyone knows it right away.*

K2 (II) *Everyone turns their head, so the pressure raises… that there is something wrong.*

(Krakow, GP 1).

There were also instances of justifying the pressure put on students. Some of the students believed that due to the constant criticism they became more resistant to stress. Such mental strength is very much needed in the medical profession.

K (III) *( … ) Because we meet many diseases, we meet people who are simply sick, and these studies show us how to treat people, how people come out of different states. But there are also many such moments that are simply overwhelming and sad, and it seems to me that maybe that’s why we are, I don’t know, subjected to such greater, greater pressure, we must be more resistant to stress, because we just seem to see more such sad situations ( … )*

(Krakow, GP 2).

The students pointed out that they are treated the best by the younger doctors who understand and still remember what they went through during their studies. They mentioned, however, that some lecturers take their frustrations out on students for what happened to them during their university years. One of the respondents stated that the result of the mistreatment of students is that, as doctors, they will not have an appropriate empathetic approach to patients.

K (III) *It is said that doctors do not treat patients with respect often, but I have the impression that in our studies we are also very often treated with disrespect ( … ). So, no one really teaches us this respect for other people ( … ).*

(Gdansk, GP 1).

## 4. Discussion

The study shows that Polish medical students are mistreated during medical school in various ways. They experience verbal abuse, they are humiliated, insulted, criticised, shouted at for minor mishaps, and the lecturers also make indecent comments directed at the students. Studies in the 1960s discussed the humiliation experienced by American students [12]. More recently, in a qualitative study, Pitkala and Mantrant stated that feelings such as anxiety, stress, and fear of humiliation are common in hospital culture [17]. Also, many quantitative studies show that medical students experience belittlement and humiliation [18,19,20,21,22,23,24].

It is worth emphasising that not all students in the present study reported experiences of verbal abuse from their lecturers, and this could be for several reasons. The first, and most obvious, is that it may not have happened to them. Second, such situations might have happened to them, but they do not care, or the incidents were not important enough for them to remember. The perception of whether a given behaviour is abuse may vary. Third, as the medical community is very tight knit, the students who are part of this community, may not want, using Goffman’s [37] terminology, to let the focus group facilitator see ‘backstage’. During medical studies, the students are taught not only values, but also a new way of thinking and even a way of speaking [38]. Some authors consider the habitus concept by Bourdieu [39] as a useful concept to understand the assimilation process of future physicians into medical culture [40,41]. It is the habitus that gives the medical community a strong sense of professional solidarity [41] and not everyone might find it appropriate to share stories of medical student abuse with a the focus group facilitator.

Hierarchy in the medical community is extremely important because relations between colleagues are based on the unquestionable subordination of people in lower positions by those in higher positions. Such subordination is justified by the urgency of the medical tasks [11]. Medical students are placed very low in this hierarchy, which makes them even more susceptible to mistreatment [42]. One of the forms of abuse reported by medical students in a survey by Silver and Glicken [19] were intentional neglect or a lack of communication. In this study, we refer to this kind of abuse as disregard. Students reported that the lecturers do not respect their time (e.g., they are late to class) but on the other hand, students are required to always be punctual. Respondents said that faculty do not understand the extenuating circumstances that may have caused an absence. Students mentioned that sometimes lecturers were not prepared for their classes. There were also situations where the clinical teachers tended to hide in hospitals, away from the students. Another Polish study indicated that the greatest disappointment among first-year medical students was the “lack of support from faculty and their lack of interest in teaching” [30] (p. 217). A study by Nagata-Kobayashi et al., among Japanese medical students, found that many of them were often neglected and disrespected by their lecturers [23]. Their article states, “commitment to teaching is not the opposite of mistreatment or abuse of students, ‘ethical’ or ‘professional’ treatment of medical students is an important factor in high-quality clinical clerkships” [23] (p. 217).

As discussed earlier, despite the limits placed on medical school admissions in Poland, students found that there are still too many students accepted especially given the complexities of the Polish educational system. Therefore, during the first year, a significant number of students drop out of their medical studies. In March 2021, students (not part of this research) recorded lecturers from two medical schools discussing different ways of lowering the number of students who pass exams; they subsequently made this recording public. This caused an uproar in the Polish media [43] and confirmed the allegations of the students from the present study. Respondents believed that the tests were not designed to check relevant knowledge, but instead, they were designed to ensure the appropriate number of failures. This is a systemic problem whereby lecturers do not specify what the students should learn and from which textbooks. Additionally, the students indicated that they must review outdated information that may be inaccurate and is consistent with the lecturer’s beliefs, whether medically correct or incorrect. Additionally, some subjects cannot be repeated, and some are too expensive to repeat, meaning that only wealthy students can pay for them. Therefore, students in their first years of their medical studies feel anxiety about whether they will be good enough to stay in medical school. This also creates a hostile environment between the students as there is competition for a place later in their medical studies. This pressure to learn, be overworked, adhere to strict deadlines, and the competition among each other can lead to the erosion of compassion and empathy.

Students often felt that the grades awarded to them were inadequate and unfair when compared to their knowledge and study time. This sense of unfairness during assessments was also reported by students from surveys conducted in other countries [19,20,21].

Most of the students felt that there was no point in reporting mistreatment. In one of the focus groups, the student even told a story about being intimidated by the lecturer if incidents were reported. The lack of willingness by medical students to report such treatment may be the result of Polish culture, which views informants very negatively. Reluctance to reporting has historical roots in Poland, and children are taught from an early age that it is not good to report someone else’s bad behaviour [44]. Similarly, studies from other countries also show that there is reluctance among medical students to report mistreatment. Research from Japan shows that mistreatment was not reported by 91.5% of medical students [23]. Research from the United States indicates that 69% of medical students do not report the instances of abuse which takes place during their internal medicine clerkship [24]. In Poland, further research is needed to quantify the number of experiences of abuse that are reported and to identify the reasons for the reluctance to reporting. Wolf et al. [21] lists the reasons why mistreatment may not be reported by medical students: (1) failure to recognise an incident as abuse; (2) fear of personal harm and retaliation; (3) the desire to conform and to please superiors; (4) fear of endangering their academic position, future training and job prospects; (5) students expect mistreatment to be a part of their education; (6) lack of a formal mechanism for reporting abuse; and (7) lack of assurance that an investigation will be conducted.

Students noted the negative consequences of being mistreated at medical school. They pointed out that self-esteem decreases during their studies, that they live under stress, and that very stressful situations can leave a mark on the psyche for a long time. This finding falls in line with initial research done in the 1950s and 1960s that showed that medical education is related to stress and anxiety [12,13].

Furthermore, research shows that medical students suffer from depression more often and have a range of mental disorders when compared to the general population [45]. No student in this current study has directly linked depression with experienced mistreatment, although the topic of depression as a result of medical education has appeared in different discussions. It would be logical to assume that mistreatment may be one of the factors contributing to the depression of medical professionals. This requires further research as a physician’s mental health can affect their professionalism, empathy, and humanitarian attitudes [46].

One study has already linked the repeated mistreatment of medical students to a high likelihood of burnout [26]. Burnout is “long-term reaction to stress characterised by emotional exhaustion, depersonalisation, cynicism, and feelings of decreased personal accomplishment” [47] (p. 2). Although the students in this study discussed burnout, they did not link it to the mistreatment they experienced. Similar to depression, we could logically assume that experiences of mistreatment may be one of the factors contributing to the development of burnout. In Poland, the percentage of medical students suffering from burnout syndrome increases with the duration of their studies [31]. This topic also seems to be important in future research, as burnout of can have serious consequences for public health [48].

One of the students drew attention to the fact that because they are treated with disrespect during their medical education, they do not learn to respect other people, which then means that they may treat their own patients with disrespect. It is worth noting that within the medical community, disrespect is common, and it also applies to relationships between colleagues. Mobbing is a significant problem among healthcare professionals. Mobbing is the word used in the Polish Labor Code (see: article 943 paragraph 2) [49]; similar to workplace bullying. Research indicates that one in every eight employees in the medical community in Poland has experienced mobbing [50]. Sometimes, the victim of mobbing is not only the employee who is harassed but also the patient who is sometimes used a ‘tool’ of the mobber [50]. It could be assumed that such interpersonal problems have their beginnings in the processes associated with academic education because of a hidden and informal curriculum. Students then go on to adopt the practice, culture, and patterns of behaviour from their more experienced colleagues.

Students also noticed that there are some lecturers who reproach them for what happened to them during their own studies. The same observation was made by Key who stated that lecturers treat students badly because they themselves were treated badly [9]. In his opinion, medical culture is associated with a traumatic de-idealisation that is passed down from generation to generation [9].

As early as 1990, Silver noticed that bad treatment was sometimes ignored and often even explained as for the student’s own good [15]. He wrote, “Everyone needs to have this experience. It’s good for them. It will help them to be better doctors.” [15] (p. 309). Other researchers also wrote about a similar belief widespread in the medical community, that mistreatment has useful functions [51,52]. Some of the students in this study also excused mistreatment. Students thought that this was a way to strengthen their psyche and make them more resistant to the stress that they will experience in their future careers. In social psychology, the mechanism of psychological rationalisation has been described and is used in order to survive severe initiations. The use of rationalisation causes neutralization not only to one’s own bad experiences but also to similar problems experienced by other people [53].

The present study is not without limitations. It is important to note that the mistreatment of medical students was neither the main nor even a secondary topic of the research. None of the questions in the interview script covered this topic. The issue of mistreatment appeared in all focus groups but because it was not the main objective of the research, it was not explored further. Therefore, the issue of physical abuse, sexual abuse, or gender discrimination did not arise. Even if asked directly, we do not know if the students would have reported these forms of abuse. Another limitation of the study that could affect the results was the fact that more than half of the students (48) were from junior years (II-III), which means that they have not yet started their clinical practice. Younger students might not have had the same experiences as those in their senior years, including the levels of mistreatment. Further research on this issue should therefore be carried out with senior students. The students in this study were reimbursed for their participation which could have influenced their responses as, for example, they may have embellished their accounts to make them sound more interesting. An additional limitation that could influence the results was the fact it was not possible to organise groups in such a way that the students did not know each other (for example, some of the students in a focus group in a particular city knew each other as they study together) so, they may have felt self-conscious about their own mistreatment stories and would not have wanted to share their experiences with the group. On the other hand, the students knowing each other may have had a positive effect in that the students were more likely to be truthful about their mistreatment, because others could verify their stories. An additional limitation of the study was its qualitative nature. Based on this research, we can only indicate that the problem of medical students’ mistreatment is present in Poland. However, we can neither show its scale nor the existing dependencies, for example, between socio-demographic variables and mistreatment.

This study also has its strengths. To our knowledge, the present study is the first in Poland to explore, in such detail, the issue of mistreatment of medical students. The concept of ‘medical student abuse’ appears marginally in Polish literature. In all of the focus groups, at least some of the students trusted the focus group facilitator to be open enough to talk about mistreatment experiences. The fact that the topic was not suggested but appeared spontaneously in all focus groups demonstrates that mistreatment is a problem of high significance for students, and this indicates the need for further research. Due to its qualitative nature, the study also shows the students’ perspectives.

Further research should be carried not only among the students to assess the scale of mistreatment, but also among lecturers to reveal to the causes of mistreatment. Perhaps, a simple solution of increasing the number of staff or lowering the student-to-lecturer ratio may improve the situation. However, it is worth noting that mistreatment cannot be justified by poor working conditions, low salary and heavy workload.

## 5. Conclusions

In Poland, as noted in some other countries, medical students face mistreatment during their education. The following topics appeared in the focus group discussions: verbal abuse, disregard, and making it difficult to pass. Although the study showed that some students do not see the point in reporting the mistreatment, they do acknowledge its negative consequences on themselves. The mistreatment of medical students must be prevented in order to preserve their mental health and so that the medical education they receive shapes them into professionals who care for and respect their patients.

The phenomenon of the mistreatment of medical students certainly requires more attention, particularly in Poland. This is the first study in Poland to detail the abuse that medical students face. At present, there is a lack of public discussion on this subject. As such, an information campaign needs to be developed and aimed towards the faculty of medical schools about the destructive consequences of the mistreatment of students. Further, the development of a systemic solution is needed to counteract such organizational pathologies.

There is an institution known as the Student Ombudsman in Poland that intervenes in cases where the rights of university students are violated. This organisation also undertakes numerous other preventive actions aimed at increasing the awareness of the rights and obligations of students. Due to the widespread mistreatment of students, it may be beneficial to develop an intervention at the university level (e.g., an ombudsman at every university) in the event that the students’ rights are violated by their lecturers. These interventions may be both preventative and corrective, particularly in the case of those universities where the incorrect attitudes of lecturers towards their students are part of the organisational culture. Further, allocating more funds and improving the infrastructure of medical education in Poland may have a positive impact on medical education.

## Data Availability

The interview transcripts from the focus groups and the script in Polish are publicly available in the Repozytorium Danych Społecznych on the website: https://rds.icm.edu.pl/dataset.xhtml?persistentId=doi:10.18150/UFEA7T (accessed on 20 November 2021).
